# Histamine-Induced Conjunctivitis and Breakdown of Blood–Tear Barrier in Dogs: A Model for Ocular Pharmacology and Therapeutics

**DOI:** 10.3389/fphar.2019.00752

**Published:** 2019-07-09

**Authors:** Lionel Sebbag, Rachel A. Allbaugh, Amanda Weaver, Yeon-Jung Seo, Jonathan P. Mochel

**Affiliations:** ^1^Department of Veterinary Clinical Sciences, College of Veterinary Medicine, Iowa State University, Ames, IA, United States; ^2^Department of Biomedical Sciences, College of Veterinary Medicine, Iowa State University, Ames, IA, United States; ^3^Lloyd Veterinary Medical Center, College of Veterinary Medicine, Iowa State University, Ames, IA, United States

**Keywords:** albumin, blood–tear barrier, conjunctivitis, dog, histamine, ocular surface

## Abstract

Conjunctival inflammation disturbs the blood–tear barrier and thus affects the tear film stability and composition. We aimed to develop a non-invasive and reliable method to induce conjunctivitis in dogs, a large animal model for translational work on ocular surface disease in humans. Six beagle dogs underwent a randomized, vehicle-controlled, balanced crossover trial—on six separate days, one eye received topical artificial tears (vehicle), while the other eye received one of six concentrations of histamine solution (0.005–500 mg/ml). At sequential times after eyedrop administration, a conjunctivitis score was given to each eye based on the degree of palpebral and bulbar conjunctival hyperemia and chemosis, ocular pruritus, and discharge. Total protein content (TPC) and serum albumin were quantified in tear fluid at baseline and 20 min. Additionally, 13 dogs presenting for various ophthalmic diseases with associated conjunctivitis were examined. Experimentally induced conjunctivitis developed rapidly (<1 min) following topical histamine administration and lasted for 1–3 h (four lowest doses) to 6–8 h (two highest doses). The severity of conjunctivitis was dose-dependent. Histamine was overall well tolerated, although transient blepharitis, aqueous flare, and ocular hypertension occurred in a few dogs receiving histamine ≥375 mg/ml. TPC and serum albumin levels increased in tears of eyes receiving histamine ≥1.0 mg/ml, being significantly higher than vehicle and baseline in eyes receiving histamine ≥375 mg/ml. Lacrimal albumin levels were also increased in 13 dogs with naturally acquired conjunctivitis, up 2.7–14.9 fold compared to contralateral healthy eyes. Histamine-induced conjunctivitis represents a robust model for translational work on the ocular surface given the low cost, non-invasiveness, self-resolving nature, ability to adjust the duration and severity of the disease, and shared features with naturally occurring ocular diseases. Histamine solutions of 1, 10, and 375 mg/ml induce mild, moderate, and severe conjunctivitis in dogs, respectively. Leakage of serum albumin in tear fluid of eyes with conjunctivitis suggests a breakdown of the blood–tear barrier.

## Introduction

Conjunctivitis, or inflammation of the vascularized mucous membrane lining the inside of the eyelids, anterior sclera, and (when present) the nictitating membrane, is a common ocular surface disease in both humans and veterinary patients (Azari and Barney, 2013; Maggs, 2018). In addition to well-recognized etiologies (e.g., infectious, allergic, toxic/irritative, immune-mediated), conjunctivitis frequently develops as a bystander to most adnexal and ocular diseases, such as blepharitis, keratitis, uveitis, and glaucoma (Azari and Barney, 2013; Maggs, 2018). Regardless of the cause, conjunctivitis is debilitating to patients due to ocular discomfort, redness, and discharge, as well as the potential development of conjunctival scarring, fornix foreshortening, or symblepharon in severe or untreated cases. Further, conjunctivitis compromises the tear film homeostasis and thereby contributes indirectly to ocular surface damage. Changes in tear composition result from a loss of secretory function and numbers of mucin secreting goblet cells (Contreras-Ruiz et al., 2013), but can also be linked to the disruption of the blood–tear barrier—a critical yet poorly understood mechanism (van Delft et al., 1997). Indeed, conjunctivitis increases vascular permeability and results in leakage of plasma compounds into the tear film, as exemplified by human patients with conjunctivitis and dry eye who were noted to have significantly greater serum albumin in tears compared to healthy controls (Zavaro et al., 1980; van Bijsterveld and Janssen, 1981; Janssen and van Bijsterveld, 1986; Li et al., 2010), and dogs with spontaneous keratoconjunctivitis sicca for whom the clinical signs of conjunctivitis were positively correlated with tear levels of serum proteins (Fullard et al., 1995).

Despite the prevalence of conjunctivitis, there is limited knowledge about the disease impact on the ocular surface in clinical patients. Are tear film composition and quality affected by conjunctivitis-induced breakdown of the blood–tear barrier? Is there an impact on the pharmacokinetic profile of drugs on the ocular surface? Do medications administered systemically reach the tear film compartment at higher concentrations? Such knowledge can be gained from experimentally induced models of conjunctivitis in animals, such as intraperitoneal injection of ovalbumin in guinea pigs (Shii et al., 2010) and topical administration of dust mite allergens in dogs (Lourenço-Martins et al., 2011). However, a delayed response occurs with ovalbumin (6 h from antigen exposure to conjunctival pathology), and dust mite allergens only cause conjunctivitis in individuals already sensitized to this antigen. In contrast, the use of topical histamine is promising as it causes conjunctivitis in a nonspecific and rapid manner (Takahashi et al., 2010) and the compound is a potent inflammatory mediator that is associated with various disorders such as allergy, inflammation, autoimmune conditions, and possibly cancer (Branco et al., 2018). Takahashi and colleagues used topical histamine in guinea pigs and quantified the extravasation of Evans Blue to demonstrate vascular permeability in the conjunctiva (Takahashi et al., 2010). The authors focused on a single dose of histamine and did not assess the safety of the drug, disease severity, or disease duration. In the present study, the model of histamine-induced conjunctivitis was fine-tuned and perfected: we investigated a diverse range of histamine concentrations, performed serial ophthalmic and physical examinations to assess safety, and described in detail the clinical and biochemical changes observed at each dose. The dog is a preclinical species of choice for modeling human ocular diseases as the canine ocular anatomy is more similar to humans than routine laboratory species (Vézina, 2013); dogs share similar environmental stressors with people, and spontaneously occurring ocular surface diseases are common in this species. We are confident that this translatable *in vivo* model of conjunctivitis will guide future studies to gain a deeper understanding of the disease, elucidating the impact of conjunctivitis on drug pharmacokinetics, tear film dynamics, metabolomics, and proteomics, among others.

## Materials and Methods

### Experimentally Induced Conjunctivitis in Dogs

#### Animals

Six beagle dogs (1.5–2.0 years, 7.7–10.1 kg) were used in the study. The gender and neuter status was the same for all subjects (female spayed), as sex hormones are known to be key regulators of vascular tone in various organs, including the eye (Schmidl et al., 2015). All dogs were confirmed to be healthy based on complete physical and ophthalmic examinations, including slit-lamp biomicroscopy (SL-17; Kowa Company, Ltd., Tokyo, Japan), indirect funduscopy (Keeler Vantage; Keeler Instruments, Inc., Broomall, PA, USA), rebound tonometry (TonoVet; Icare Finland Oy, Espoo, Finland), Schirmer tear test-1 (STT-1; Eye Care Product Manufacturing LLC, Tucson, AZ, USA), and fluorescein staining (Flu-Glo, Akorn, Inc., Buffalo Grove, IL, USA). The dogs were group-housed in kennels with ambient temperature maintained at 18–24°C and lights automatically turned on/off at 06:00/18:00. The study was approved by the Institutional Animal Care and Use Committee of Iowa State University (log # 2-18-8704-K) and adhered to the Association for Research in Vision and Ophthalmology (ARVO) statement for the Use of Animals in Ophthalmic and Vision Research.

#### Topical Histamine and Vehicle Solutions

Histamine ophthalmic solutions were formulated by mixing histamine powder (Histamine dihydrochloride, FCC grade, Arcos^®^ organics, Geel, Belgium) in 1.4% polyvinyl alcohol lubricating eye drops (Artificial tears solution, Rugby, Rockville Center, NY, USA) using a sterile manner under a laminar flow hood. The pH of each solution was tested with a pH meter (B-212 Twin compact pH, Horiba, Kyoto, Japan) and adjusted to 6.5 by adding 1% sodium hydroxide (prepared from granules mixed with sterile water), one drop at a time until the target pH was reached. The following 12 concentrations of histamine solution were compounded into 15-ml sterile eyedropper bottles (Steri-dropper^®^, Medi-Dose^®^, Ivyland, PA, USA) by the pharmacist at Iowa State University’s Lloyd Veterinary Medical Center: 0.001, 0.005, 0.01, 0.0375, 0.1, 0.5, 1.0, 10, 100, 375, 500, and 1,000 mg/ml. The vehicle solution consisted of artificial tears solution adjusted to pH of 6.5 with a minute amount of 1% sodium hydroxide (< 5 drops in 15-ml bottle). Histamine and vehicle solutions were used within 24 h of preparation and kept in the dark at room temperature (18–24°C) before use and in between experiments.

#### Experimental Design

##### Pilot Study to Assess Tolerability and Appropriate Histamine Concentrations

A pilot study was conducted to assess the tolerability of histamine solutions in dogs (local and systemic) and the most appropriate concentrations to select for the crossover trial. Each of the 12 eyes (*n* = 6 dogs) was randomly allocated to one of the 12 histamine concentrations (Excel, Microsoft, Redmond, WA, USA). A single drop of histamine solution was instilled onto the ocular surface, followed by slit-lamp biomicroscopy and conjunctivitis scoring at 1, 3, 5, 7, 10, 15, 20, 25, 30, 40, 50, 60, 90, 120, 180, 240, 300, 360, and 420 min. Additionally, the following ocular and physical parameters were recorded at 1, 10, 20, 30, 60, 120, and 240 min post-histamine administration: intraocular pressure, aqueous flare grading, body temperature, heart rate, respiratory rate, respiratory efforts, capillary refill time, and indirect blood pressure (Doppler model 811-B, Parks Medical Electronics, Las Vegas, NV, USA).

##### Balanced Crossover Vehicle-Controlled Trial

Six histamine solutions were selected for the crossover trial based on good tolerability and diversity of conjunctivitis scoring (pilot study, data not shown). The six histamine solutions are listed as follows: H_1_ = 0.005 mg/ml, H_2_ = 0.1 mg/ml, H_3_ = 1.0 mg/ml, H_4_ = 10 mg/ml, H_5_ = 375 mg/ml, and H_6_ = 500 mg/ml. For each dog, one eye received histamine solution (random selection by coin-toss) while the other eye received vehicle solution, and this order was kept consistent throughout the study. Each dog received all six histamine solutions over six consecutive days, using one solution per day (once in the morning) in a balanced crossover trial (**Supplementary Figure 1**). In each dog, ocular and physical parameters were recorded at selected times (similar to pilot study), while tear collection and conjunctivitis scoring were performed as outlined below:

- **Tear collection**: At baseline and at 20 min post-eyedrop administration, tear fluid was collected from both eyes and total protein content (TPC) was analyzed as previously described (Sebbag et al., 2018b). Briefly, a standardized Schirmer strip was inserted into the ventrolateral conjunctival fornix until the 20-mm mark was reached. Each wetted Schirmer strip was placed into a 0.2-ml tube (pre-punctured at its bottom with an 18-gauge needle), secured into a 2-ml tube with adhesive tape, and centrifuged at 3,884 × *g* for 2 min (Mini Centrifuge, VWR International, Radnor, PA, USA). After estimating the volume of extracted tear fluid with a pipette, each tear sample was diluted 1:3 with phosphate-buffered saline (PBS 1X, Gibco^™^, Thermo Fisher Scientific, Inc., Waltham, WA, USA). TPC was calculated with Direct Detect^™^ infrared spectrometer and expressed in mg/ml after adjusting for the threefold dilution of each sample. Subsequently, the residual tear fluid was diluted fourfold with diluent provided with the albumin ELISA kit (Serum albumin ELISA kit, Life Span Biosciences, Inc., Seattle, WA, USA). Serum albumin and various cytokines/chemokines/growth factors (Canine Procarta Plex^™^ 11-plex immunoassay, Cat No EPX11A-50511-901, Thermo Fischer Scientific, Waltham, MA, USA) were quantified in each tear sample following the manufacturer’s protocol.
- **Conjunctivitis score**: At baseline and at selected times post-eyedrop administration (similar to pilot study), one examiner (LS) who was masked to which eye received histamine or vehicle solution performed slit-lamp biomicroscopy of both eyes to determine a conjunctivitis score at each time point. The conjunctivitis score was calculated as the sum of the following categories, each graded on a three- to four-point scale (**Supplementary Table 1**): hyperemia, chemosis and follicles of the palpebral and bulbar conjunctiva, conjunctival discharge, and ocular pruritus (Uchio et al., 2008; Eaton et al., 2017).

### Naturally Acquired Conjunctivitis in Dogs

Thirteen dogs presenting to Iowa State University’s Lloyd Veterinary Medical Center (ISU-LVMC) for clinical ophthalmic disease with associated conjunctivitis were enrolled. A verbal informed consent was obtained from all owners, which was sufficient for the hospital’s ethics committee given that Schirmer tear test is part of routine clinical care. Dogs were examined by a board-certified veterinary ophthalmologist (LS or RA) for diagnosis and treatment of various ocular complaints including corneal ulceration, uveitis, and glaucoma. Details of the dogs (breed, sex, age) and their clinical diagnosis are presented in **Table 1**. Tear collection and albumin analysis were performed in both eyes of each dog as described above.

**Table 1:** Patient information and tear albumin concentrations in the affected and unaffected eyes of dogs presented to the Ophthalmology service at Iowa State University’s Lloyd Veterinary Medical Center with various ocular diseases.

Case #	Patient information	Ocular disease	Conjunctivitis score (affected eye/unaffected eye)	Tear albumin concentration in affected eye (mg/ml)	Tear albumin concentration in unaffected eye (mg/ml)	Ratio affected vs. unaffected eye
1	9 yo FS Yorkshire terrier	Keratoconjunctivitis sicca	4/0	3.50	1.22	2.87
2	9 yo MC French Bulldog	Corneal ulcer (superficial), Eyelid mass	6/1	2.44	0.80	3.05
3	4 yo MC Shih Tzu	Corneal ulcer (superficial), Distichia	3/1	1.10	0.38	2.89
4	6 yo MC English Bulldog	Spontaneous chronic corneal epithelial defect	8/0	9.98	1.67	5.99
5	12 yo FS Shih Tzu	Corneal ulcer (stromal)	5/0	3.51	0.84	4.18
6	12 yo FS English Springer Spaniel	Cataract, Lens-induced uveitis	5/1	3.51	0.94	3.72
7	3 yo MC Border Collie	Uveitis (blastomycosis)	7/0	13.96	3.06	4.57
8	12 yo FS mixed breed	Uveitis, Glaucoma (secondary)	5/0	1.80	0.24	7.52
9	4 yo MC Japanese Chin	Glaucoma (primary)	6/1	1.82	0.68	2.67
10	8 yo FS Beagle	Orbital cellulitis	5/0	1.93	0.13	14.86
11	7 yo FI Coonhound	Glaucoma (primary), end-stage	6/0	17.95	1.72	10.42
12*	5 yo MC Pitbull	Spontaneous chronic corneal epithelial defect	7/1 1/1	6.30 1.74	1.77 1.74	3.55 1.0
13*	4 yo FS Beagle	Uveitis (blastomycosis)	6/0 2/0	1.52 0.37	0.42 0.34	3.60 1.09

*Cases 12 and 13 were examined twice: findings at the initial visit are shown in the first row, while findings following treatment of the ocular disease are depicted in the second row. yo, year old; FS, female spayed; MC, male castrated; FI, female intact.

### Data Analysis

Normality of the data was assessed with the Shapiro–Wilk test. The one-way ANOVA and Tukey *post hoc* test was used to compare the six histamine doses for i) duration of conjunctivitis, determined as the time for conjunctivitis score to return to zero, and ii) severity of conjunctivitis, determined by the area under the curve of conjunctivitis score from 0 to 180 min. Within each histamine dose, the one-way repeated measures ANOVA and Bonferroni *post hoc* test was used to assess differences in conjunctivitis scores between time points. The Student t-test was used to evaluate differences between vehicle and histamine-treated eyes for TPC and albumin levels, both at baseline and at 20 min following eyedrop administration. Further, a ratio was calculated for TPC and albumin levels for each histamine dose as follows: (Concentration histamine 20 min/histamine baseline)/(Concentration vehicle 20 min/vehicle baseline). This ratio describes the percentage change in protein levels between baseline and 20 min post-induction of conjunctivitis, taking into account the variability inherent to the tear collection method itself by adding the vehicle-treated eyes as the denominator (Sebbag et al., 2018b). The one-way ANOVA and Tukey *post hoc* test was used to assess differences in protein ratios and levels of cytokines/chemokines/growth factors in tears of different groups. In dogs with naturally acquired conjunctivitis, differences in lacrimal concentrations of albumin between affected and unaffected eyes were analyzed with the Mann–Whitney test. Statistical analysis was performed using SigmaPlot version 14.0 (Systat Software, Point Richmond, CA), and values *P* < 0.05 were considered statistically significant.

## Results

### Experimentally Induced Conjunctivitis in Dogs

#### Clinical Features of Conjunctivitis

**Figure 1:**
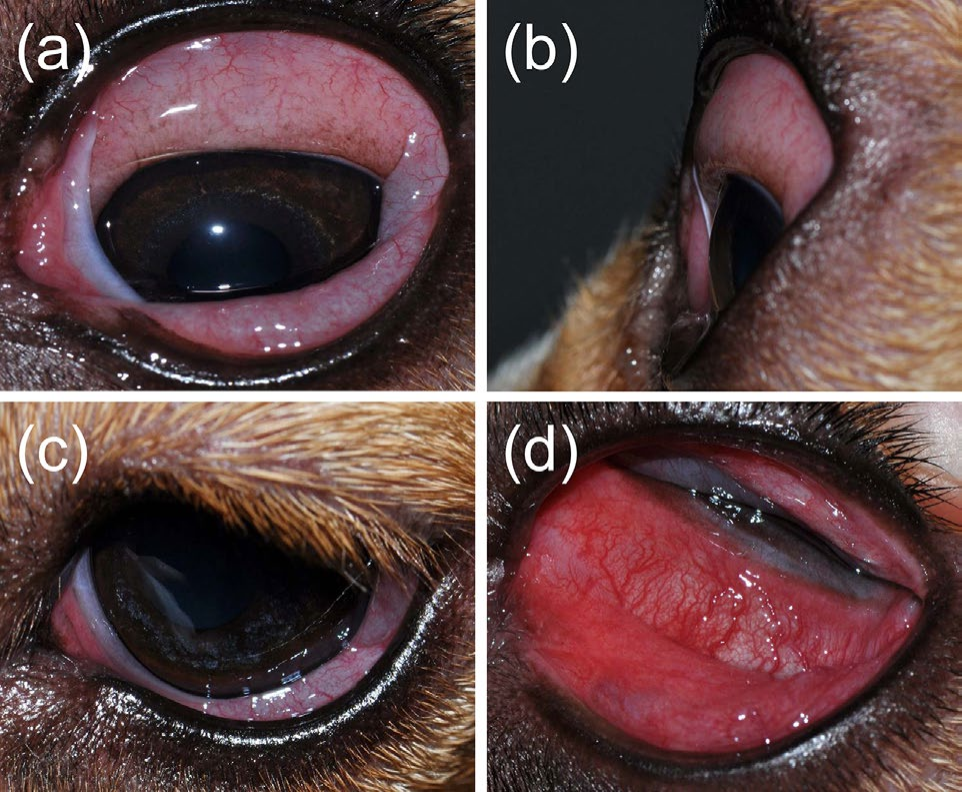
A comprehensive evaluation of the canine conjunctiva is facilitated by examination of the ocular surface at different angles, including a front view **(A)**, side view **(B)**, top view **(C)**, and globe retropulsion with lower lid retraction **(D)**. The latter permits visualization of the palpebral conjunctiva and nictitating membrane.

**Figure 2:**
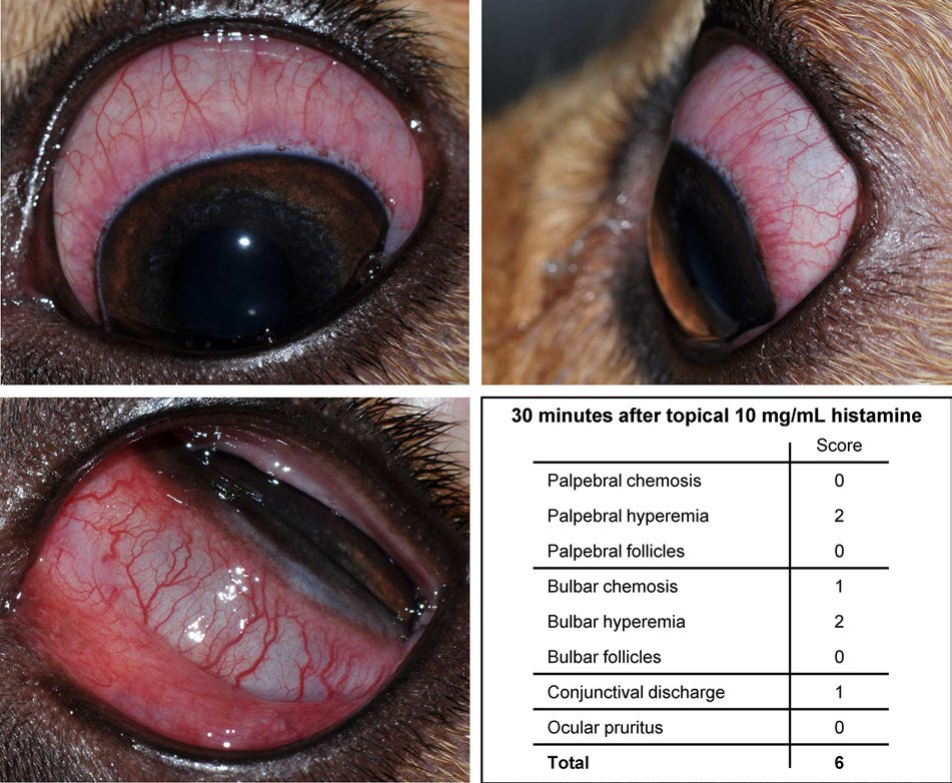
Photographs of the left eye in a dog, 30 min following topical administration of 10 mg/ml histamine solution. The overall conjunctivitis score was 6, based on the absence/presence and degree of follicles, hyperemia, and chemosis of both palpebral and bulbar conjunctiva, conjunctival discharge, and ocular pruritus.

**Figure 3:**
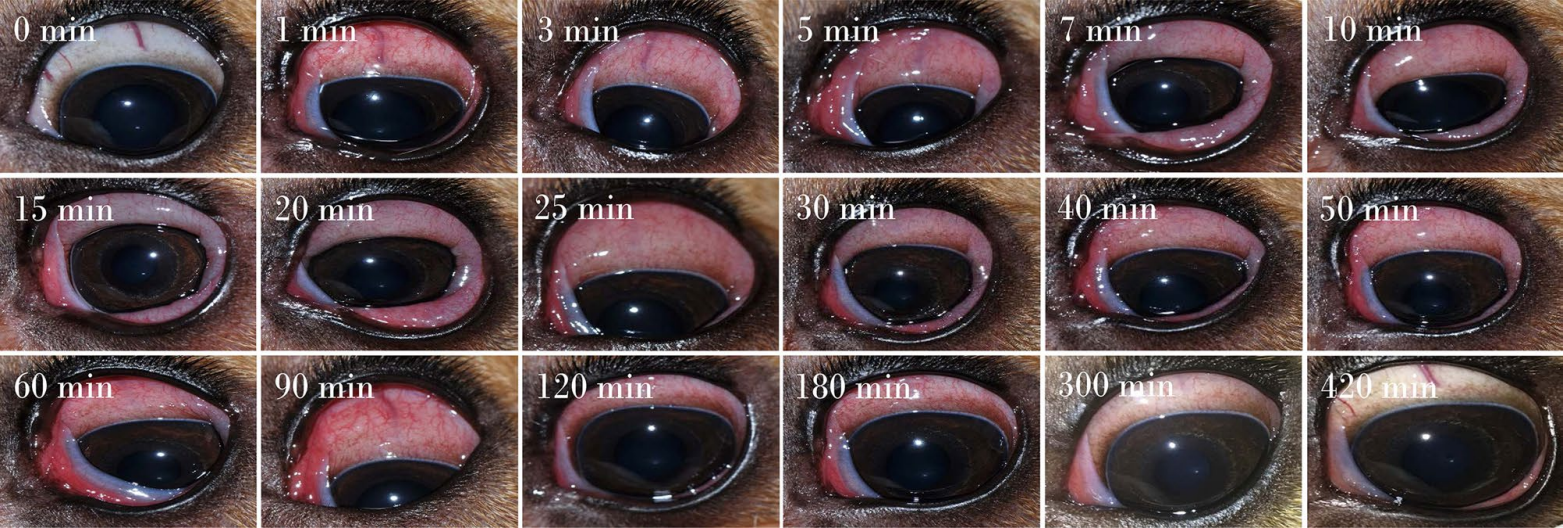
Development and progression of conjunctivitis in a representative canine eye receiving topical 375 mg/ml histamine solution. Conjunctivitis developed rapidly (< 1 min), with progression of conjunctival hyperemia and chemosis for 20–30 min, and subsequent slow improvement and self-resolution by 420 min.

All eyes receiving the vehicle solution were scored at zero for all time points. In eyes receiving histamine, the ocular surface was examined from different angles to evaluate the canine conjunctiva in a comprehensive manner, including a view from the front (**Figure 1A**), side (**Figure 1B**), top (**Figure 1C**), and globe retropulsion with lower lid retraction (**Figure 1D**), which facilitated assessment of the palpebral conjunctiva and nictitating membrane. **Figure 2** shows representative photographs and conjunctivitis scoring of a canine eye at 30 min following H_4_ administration (10 mg/ml histamine solution), while **Figure 3** demonstrates the development and progression of conjunctivitis from 0 to 420 min in a canine eye receiving H_5_ (375 mg/ml histamine solution). Data were normally distributed such that results are presented as mean ± standard deviation (range).

**Figure 4:**
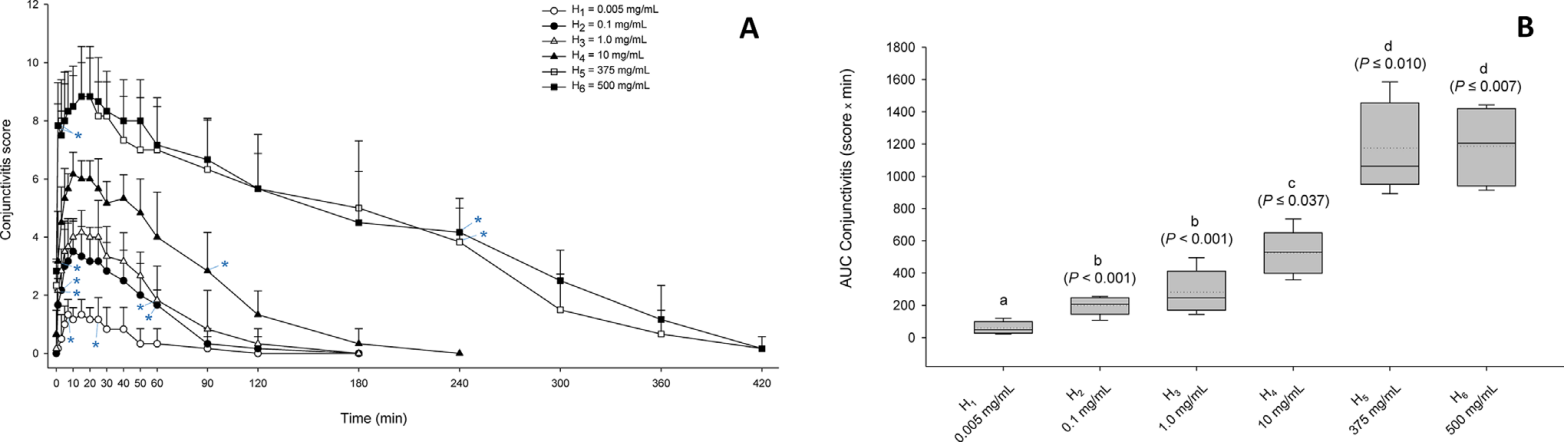
**(A)** Graphs depicting the mean + SD of conjunctivitis score over time in dogs receiving H_1_ (0.005 mg/ml; white circles), H_2_ (0.1 mg/ml; black circles), H_3_ (1.0 mg/ml; white triangles), H_4_ (10 mg/ml; black triangles), H_5_ (375 mg/ml; white squares), and H_6_ (500 mg/ml; black squares). Within the same dose, a blue asterisk (*) depicts statistical significance of conjunctivitis scoring compared to baseline (for readability, only the first and last significant time points are depicted). **(B)** Box-and-whiskers plots depicting the area under the curve of conjunctivitis score from 0 to 180 min in dogs receiving topical histamine of various concentrations. Mean and median values are shown by horizontal dotted and solid lines, respectively. First and third quartiles (25th and 75th percentiles) are represented by the lower and upper limits of the box, respectively, while the 2.5th and the 97.5th percentiles are shown as the lower and upper whiskers, respectively. The conjunctivitis severity of histamine doses with different letters differ significantly (*P* < 0.05).

Conjunctivitis developed rapidly (<1 min) for all doses (**Figure 4A**). Of note, not a single eye developed conjunctival follicles. Details of conjunctivitis scoring for each subsection (palpebral chemosis, bulbar hyperemia, etc.) is described in **Supplementary Table 2**.

The duration of conjunctivitis was statistically different among histamine doses (*P* < 0.001), ranging from 61 ± 37 min (25–120 min) for H_1_, 110 ± 36 min (90–180 min) for H_2_, 115 ± 52 min (60–180 min) for H_3_, 190 ± 45 min (120–240 min) for H_4_, 390 ± 33 min (360–420 min) for H_5_, and 400 ± 31 min (360–420 min) for H_6_. All pairwise comparisons were statistically significant (*P* ≤ 0.029) except for H_1_–H_3_ (*P* = 0.201) and H_5_–H_6_ (*P* = 0.998).

The severity of conjunctivitis was significantly different among histamine doses (*P* < 0.001), with an AUC_0–180min_ (in score _x_ min) ranging from 59.2 ± 36.8 (21.5–119) for H_1_, 200.2 ± 53.9 (107.5–254.5) for H_2_, 277.2 ± 128.3 (144.5–495.5) for H_3_, 522.3 ± 131.8 (358–735) for H_4_, 1,168.5 ± 266.4 (892.5–1,585) for H_5_, and 1,186.5 ± 242.4 (914–1,443.5) for H_6_. All pairwise comparisons were statistically significant (P ≤ 0.037) except for H_5_–H_6_ (*P* = 1.000) and H_2_–H_3_ (*P* = 0.794; **Figure 4B**).

A *post hoc* power analysis showed that *n* = 5 dogs were sufficient to detect a difference in total clinical scores of 2.2 (as observed clinically in eyes with mild *vs.* moderate *vs.* severe conjunctivitis), a standard deviation of 0.9, a power of 80%, and an α value of 0.05.

#### Tolerance

**Figure 5:**
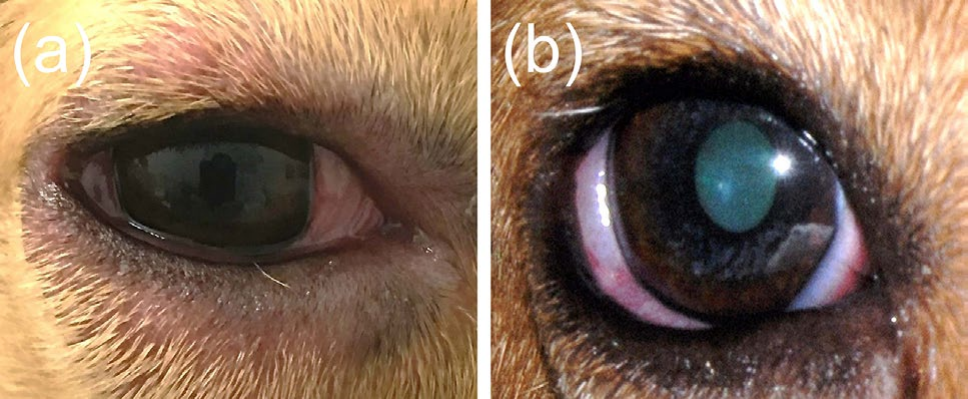
Blepharitis **(A)** and miosis **(B)** in a dog that received high dose of histamine ophthalmic solution (500 mg/ml).

Locally, histamine administration was very well tolerated in dogs except for transient adverse effects noted with H_5_ and H_6_: i) Blepharitis, manifested by mild blepharedema and erythema (**Figure 5A**), was noted in 1/6 dogs receiving H_5_ and 2/6 dogs receiving H_6_, developing within 10–30 min of histamine administration and self-resolving within 3 h. ii) Aqueous flare, subtle in intensity (trace to 1+), was noted in 2/6 dogs receiving H_5_ and 6/6 dogs receiving H_6_, developing within 25–90 min and self-resolving within 1–3 h. Aqueous flare was commonly accompanied by miosis (**Figure 5B**). iii) Ocular hypertension, defined as IOP > 25 mmHg (Sebbag et al., 2018b), was noted in 1/6 dogs receiving H_5_ at 30 min (IOP = 32 mmHg), although it was not statistically greater than baseline IOP (*P* = 0.529) and it self-resolved within 10 min. Ocular hypertension was also noted in 3/6 dogs receiving H_6_ in which IOP was significantly higher at 30 min (23.8 ± 5.5 mmHg; *P* = 0.011) and 60 min (23.2 ± 2.6 mmHg; *P* = 0.029) compared to baseline (16.8 ± 2.6 mmHg). Importantly, no fluorescein stain uptake or corneal changes were noted for any histamine dose.

Systemically, all the vital parameters monitored were stable and not a single dog receiving H_1_ to H_6_ developed systolic hypotension (<90 mmHg). However, the systolic blood pressure dropped from 130 to 88 mmHg in a single dog receiving 1,000 mg/ml histamine during the pilot phase, and the dog exhibited transient depression and weakness until blood pressure returned to baseline 10 min later.

#### Total Protein Content and Serum Albumin Levels in Tears

**Figure 6:**
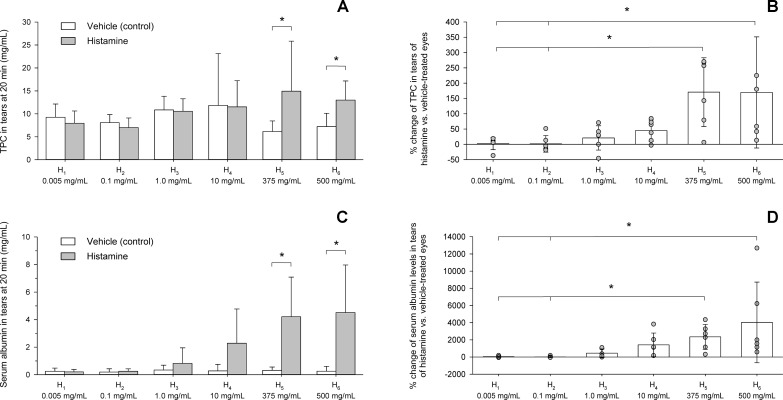
Bar charts depicting the total protein content **(A, B)** and serum albumin levels **(C, D)** in tears of six beagle dogs receiving vehicle or histamine eyedrops. An asterisk (*) indicates statistical significance (*P* < 0.05) between vehicle and histamine **(A, C)** or among histamine doses **(B, D)**.

At baseline, lacrimal TPC varied from 3.0 to 29.0 mg/ml (8.8 ± 4.0 mg/ml) and no differences were noted between vehicle and histamine-treated eyes for any dose (*P* ≥ 0.365). Twenty minutes following eyedrop administration, lacrimal TPC varied from 2.3 to 36.3 mg/ml (9.9 ± 5.6 mg/ml) and was statistically greater in histamine vs. vehicle-treated eyes for H_5_ (*P* = 0.009) and H_6_ (*P* = 0.021) but no other doses (*P* ≥ 0.310, **Figure 6A**). Mean ± SD (range) changes in TPC were 171 ± 112% (6–271%) and 170 ± 181% (13–499%) for H_5_ and H_6_, respectively, values that were significantly greater than H_1_ and H_2_ (*P* ≤ 0.036, **Figure 6B**).

At baseline, albumin levels in tears varied from 0.015 to 1.431 mg/ml (0.413 ± 0.455 mg/ml) and no differences were noted between vehicle and histamine eyes for any dose (*P* ≥ 0.394). Twenty minutes following eyedrop administration, albumin levels varied from 0.019 to 9.595 mg/ml (1.158 ± 2.098 mg/ml) and were statistically greater in histamine vs. vehicle-treated eyes for H_5_ (*P* = 0.021) and H_6_ (*P* = 0.029) but no other doses (*P* ≥ 0.106; **Figure 6C**). Mean ± SD (range) changes in albumin levels were 2,348 ± 1,454% (314–4,348%) and 4,031 ± 4,679% (583–12,689%) for H_5_ and H_6_, respectively, values that were significantly greater than H_1_ and H_2_ (*P* ≤ 0.016; **Figure 6D**).

#### Canine Chemokines/Cytokines/Growth Factors in Tears

**Figure 7:**
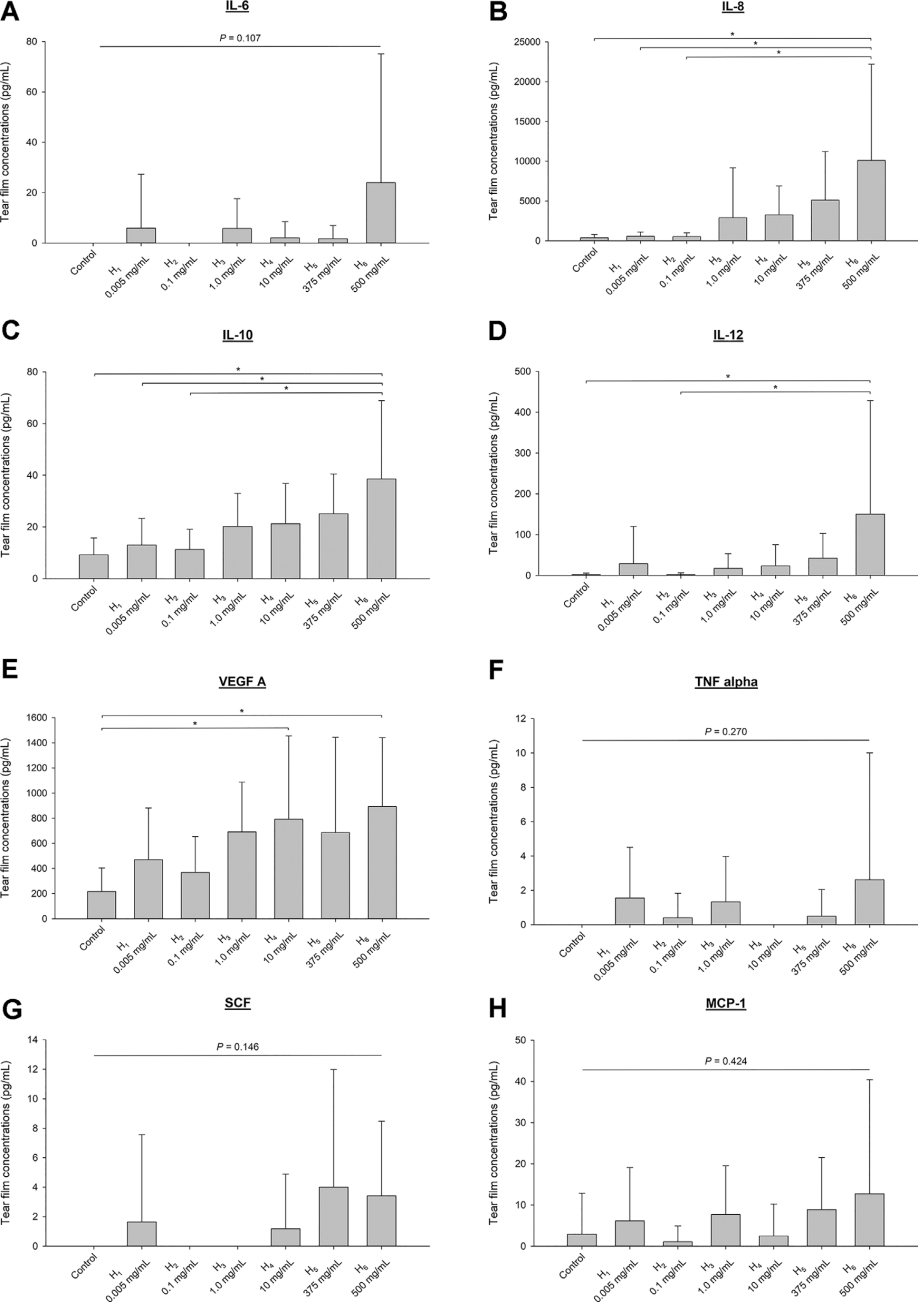
Bar charts depicting mean + SD of various chemokines, cytokines and growth factors quantified in tears of six beagle dogs receiving vehicle (vehicle) or histamine solutions: interleukin-6 (IL-6; **A**), interleukin-8 (IL-8; **B**), interleukin-10 (IL-10; **C**), interleukin-12 (IL-12; **D**), vascular endothelial growth factor A (VEGF A; **E**), tumor necrosis factor alpha (TNFα; **F**), stem cell factor (SCF; 1.3 ± 4.3 pg/ml; **G**), and chemokine monocyte chemoattractant protein-1 (MCP-1; **H**). Differences among groups are depicted by an asterisk (*) if statistically significant (*P* < 0.05).

The levels of interferon-gamma (IFNγ), interleukin-2 (IL-2), and beta nerve growth factor (NGF-β) were below limits of quantification in all tear samples. Tear concentrations of other chemokines/cytokines/growth factors, described as mean ± standard deviation (range), were as follows (**Figure 7**): 4.5 ± 19.4 pg/ml (0–147.6 pg/ml) for interleukin-6 (IL-6), 2,647.8 ± 5,680.4 pg/ml (0–25,640.2 pg/ml) for interleukin-8 (IL-8), 17.8 ± 16.3 pg/ml (0–86.8 pg/ml) for interleuekin-10 (IL-10), 30.6 ± 104.0 pg/ml (0–807.3 pg/ml) for interleukin-12 (IL-12), 532.5 ± 510.6 pg/ml (0–2,650.8 pg/ml) for vascular endothelial growth factor A (VEGF A), 0.80 ± 2.9 pg/ml (0–20.9 pg/ml) for tumor necrosis factor alpha (TNFα), 1.3 ± 4.3 pg/ml (0–24.8 pg/ml) for stem cell factor (SCF), and 5.4 ± 13.1 pg/ml (0–80.2 pg/ml) for chemokine monocyte chemoattractant protein-1 (MCP-1). Statistical differences among groups (vehicle histamine solutions) were noted for IL-8 (**Figure 7B**), IL-10 (**Figure 7C**), IL-12 (**Figure 7D**), and VEGF A (**Figure 7E**).

### Naturally Acquired Conjunctivitis in Dogs

**Figure 8:**
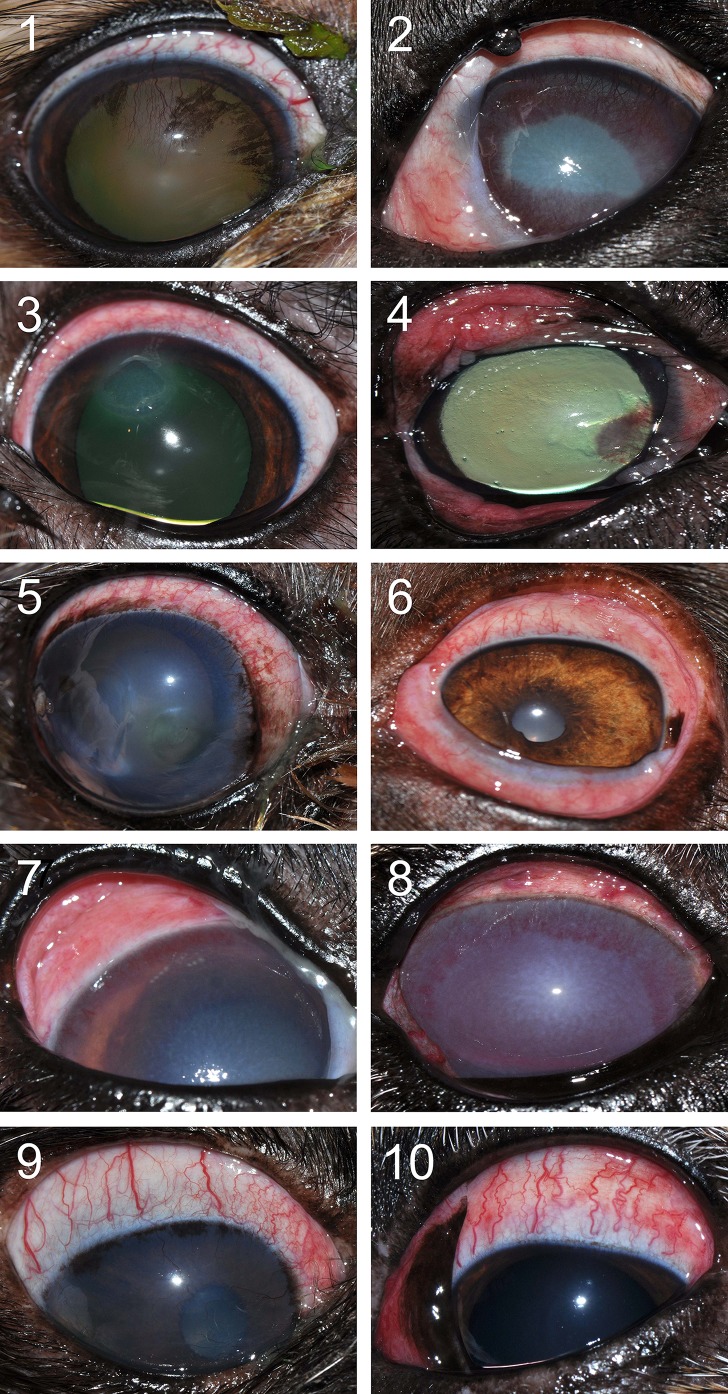
Clinical photographs of canine eyes from patients presented to the Ophthalmology Service at Iowa State University’s Lloyd Veterinary Medical Center. Conjunctivitis is present in all eyes, a condition noted concurrently to various ocular diseases. Patient case numbers are listed in the top left corner of each photograph (see **Table 1** for additional patient information).

Ocular disease was unilateral in all dogs, and spontaneous conjunctivitis was noted upon examination of all affected eyes (**Figures 8** **–** **10**). Lacrimal concentrations of albumin ranged from 1.1 to 17.95 mg/ml in affected eyes, representing a significant change by 2.67–14.86 fold (*P* < 0.001) compared to lacrimal concentrations noted in contralateral unaffected eyes (0.13–3.06 mg/ml). Re-examination of two dogs following therapy of the underlying ocular disease (cases #12–13; **Table 1**, **Figure 10**) showed reduction in lacrimal albumin concentrations concomitant with a reduction in the conjunctivitis score (**Table 1**).

**Figure 9:**
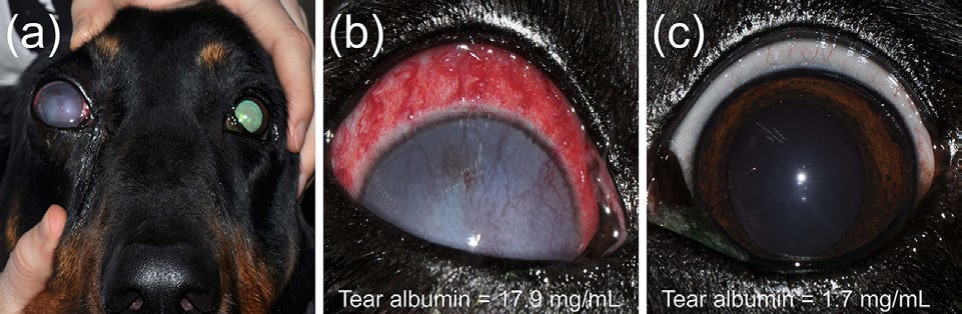
Clinical photographs of a 7-year-old female intact Coonhound dog **(A)** diagnosed with end-stage glaucoma in the right eye (case #11). Tear concentrations of albumin were much higher in the affected right eye **(B)** compared to the contralateral healthy left eye **(C)**.

**Figure 10:**
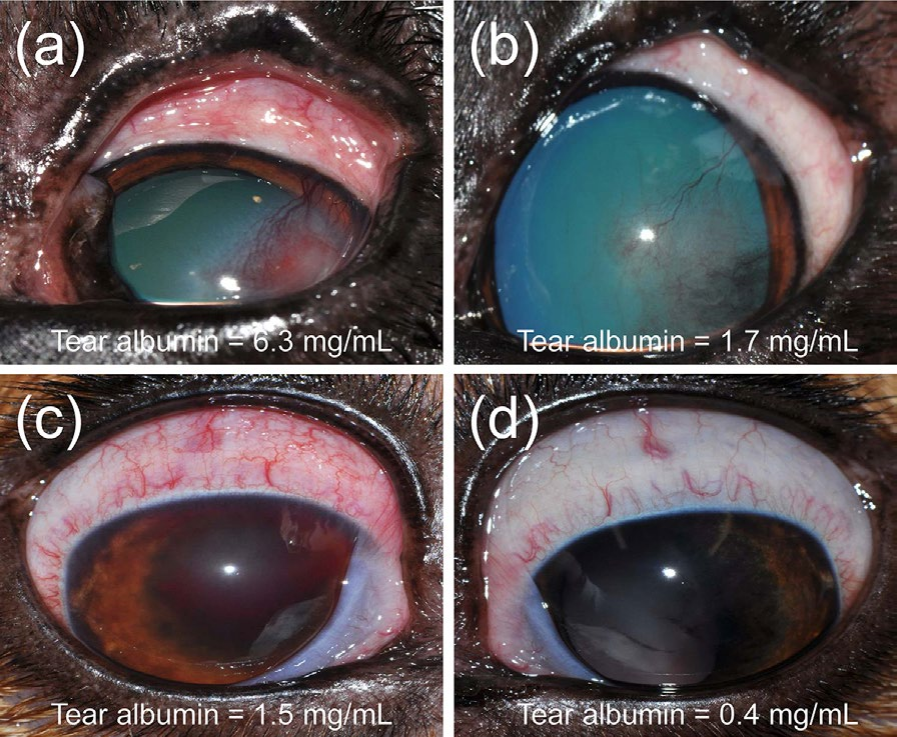
Clinical photographs of a 5-year-old male castrated Pitbull **(A, B)** diagnosed with a spontaneous chronic corneal epithelial defect (case #12) and a 4-year-old female spayed Beagle **(C, D)** diagnosed with uveitis secondary to blastomycosis (case #13). Following treatment of each ocular disease **(B, D)**, the severity of conjunctivitis was reduced and the concentration of albumin in tears was lower compared to the initial visit **(A, C)**.

## Discussion

The present study establishes a robust *in vivo* model of conjunctivitis in dogs, a translational large animal model that provides a framework for ocular surface studies in clinically relevant subjects, investigating the impact on conjunctivitis on tear film dynamics, pharmacokinetics, metabolomics, and other relevant fields. Dogs are an ideal large animal species for such translational model: not only does the canine ocular anatomy better resemble humans than small laboratory animals do (Vézina, 2013), but dogs also share similar environmental stressors to humans and conjunctivitis is a common and naturally occurring disease in this species (Maggs, 2018). As a proof of concept, our study examined 13 dogs with naturally acquired conjunctivitis and confirmed the presence of elevated albumin levels in tears of affected eyes. Similar to histamine-induced conjunctivitis, eyes with naturally occurring disease exhibited a breakdown of the blood–tear barrier regardless of the underlying etiology (e.g., corneal ulceration, uveitis, glaucoma, and orbital cellulitis). Of note, the “blood–tear barrier” is not as well defined as other ocular barriers (e.g., blood–aqueous and blood–retinal barriers) and would benefit from future anatomical and physiological studies to confirm the terminology used in the present study and in previous work (Zavaro et al., 1980; van Bijsterveld and Janssen, 1981; Janssen and van Bijsterveld, 1986; van Delft et al., 1997; Runstrom et al., 2013).

**Figure 11:**
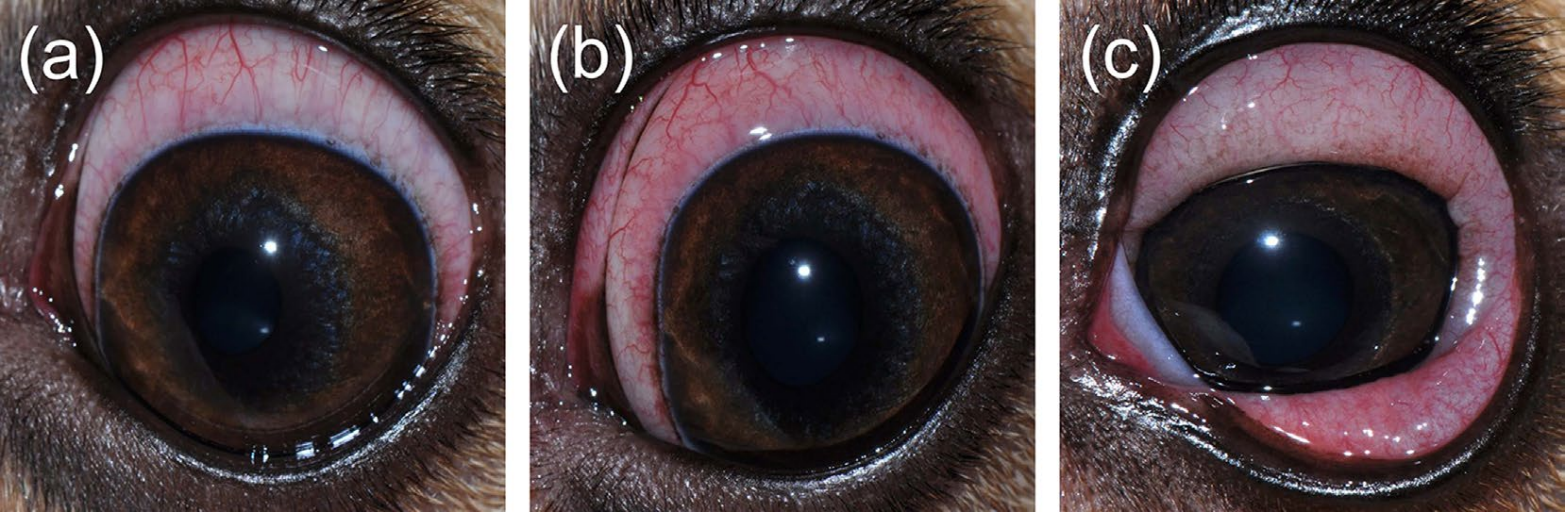
Representative clinical pictures of mild conjunctivitis (**A**; score = 3), moderate conjunctivitis (**B**; score = 6), and severe conjunctivitis (**C**; score = 9) following topical administration of histamine in dogs.

Histamine-induced conjunctivitis is not novel. The ocular use of histamine has been described in humans, guinea pigs, and rabbits as the compound is inexpensive and triggers local inflammation in a non-specific manner (Hagedoorn and Maas, 1956; Bonini et al., 1992; Takahashi et al., 2010). In dogs, we showed that histamine-induced conjunctivitis is non-invasive, self-resolving, and dose-dependent, allowing investigators to modulate the severity and duration of conjunctival inflammation by adjusting the dose of histamine solution administered. In fact, both parameters increased with histamine concentration until saturation in dose response observed at 375 mg/ml. Thus, we propose doses of 1, 10, and 375 mg/ml to induce a mild, moderate, or severe conjunctivitis in dogs, respectively (**Figure 11**). Of note, histamine is not only associated with ocular allergies but also implicated in general inflammation, autoimmune conditions, and possibly cancer (Branco et al., 2018). Ocular allergy was not the scope of the present study, as many models and detailed descriptions of the condition already exist (Bundoc and Keane-Myers, 2003; Lourenço-Martins et al., 2011).

The model presented herein provides a unique opportunity for scientists to investigate the ocular surface in health and disease. First, since topical histamine had no impact on the contralateral eye, the model is applicable for studies that compare efficacy between drug and placebo, allowing for precise measurements of a drug action given the rapid (1 min) and sustained (120–420 min) development of conjunctivitis. Second, the changes noted in tear fluid levels of TPC and albumin strongly suggest a breakdown of the blood–tear barrier. In particular, albumin represents a marker of vascular permeability and plasma leakage given it is not produced by the lacrimal gland or corneo-conjunctival tissue (Runstrom et al., 2013). Our findings are consistent with previous reports of conjunctivitis, whether experimentally induced or naturally occurring from dry eye (van Delft et al., 1997), corneal ulcers, allergies, or others (de Freitas Campos et al., 2008; Runstrom et al., 2013). The mechanism of histamine-induced disruption of the blood–tear barrier is unknown. Increased vascular permeability likely plays a role (Ashton and Cunha-Vaz, 1965; van Bijsterveld and Janssen, 1981; Takahashi et al., 2010), combined with a disruption of tight junctions between conjunctival epithelial cells due to increased contractility of actin linked to these adhesion complexes (Guo et al., 2007). The breakdown of the blood–tear barrier could be exploited for assessing the impact of conjunctivitis on drug pharmacokinetics, tear film metabolomics, and other fields. In the field of pharmacology, for instance, the vast majority of studies to date are limited to healthy subjects with intact blood–tear barriers (Hirosawa et al., 2015; Sebbag et al., 2018a; Sebbag et al., 2018b; Sebbag et al., 2018c), in whom the lacrimal drug concentrations likely under-estimate the ones noted in actual clinical patients with ocular surface inflammation (Grumetto et al., 2002). Such discrepancies likely result in inappropriate dosing, thus affecting the drug efficacy and increasing the risk of toxicity or antimicrobial resistance (Craig, 1998).

In addition to protein quantification, the present study investigated a panel of cytokines/chemokines/growth factors in tear samples of dogs. Similar to human subjects with ocular surface disease such as dry eye or vernal keratoconjunctivitis (Leonardi et al., 2009; Enríquez-de-Salamanca et al., 2010), an increase in VEGF A, pro-inflammatory cytokines (IL-8, IL-12), and anti-inflammatory cytokine (IL-10) was detected in tears of dogs with experimentally induced conjunctivitis. Analysis of other biomarkers such as acidic mammalian chitinase (Bucolo et al., 2008; Musumeci et al., 2008) would be beneficial in future studies, but the paucity of tear fluid collected in each dog limited the number of compounds that could be analyzed.

The relatively low number of dogs enrolled in the histamine experiment is a clear limitation of our work, although a *post hoc* power analysis showed that *n* = 5 dogs were sufficient to detect significant differences between histamine doses for the main study outcome (clinical severity of conjunctivitis). Furthermore, although the subjectivity of our clinical scoring may be perceived as a drawback, given that photograph-based methods are mainstream in human studies (Schulze et al., 2007; Macchi et al., 2018), the method described herein is purposely adapted to working with dogs. Indeed, i) a handheld slit-lamp greatly facilitates examination of animals for whom the skull conformation and behavior traits are poorly suited for table-mounted devices (Eaton et al., 2017), and ii) the presence of the nictitating membrane and the minimal bulbar conjunctival exposure in dogs require a “dynamic” examination of the ocular surface that is not conducive to photographic scales. The ocular adverse effects noted with high doses of histamine represent another potential limitation of the proposed model. There was minimal to absent local irritation from all doses, likely favored by adjusting the test solutions to more physiologic pH values, but histamine concentrations ≥375 mg/ml resulted in meaningful side effects in selected cases. A transient breakdown of the blood–ocular barrier likely explains the anterior uveitis (Ashton and Cunha-Vaz, 1965), while ocular hypertension may be linked to the aforementioned acute uveitis and/or episcleral venous compression from the overlying swollen conjunctiva, causing an increased resistance to aqueous outflow (Allbaugh et al., 2011; Carreon et al., 2017). Future studies are required to characterize these adverse effects in detail. Lastly, the study does not explain the mechanistic reason of breakdown of the blood–tear barrier, as we mainly focused on documenting the safety and clinical features of the model in dogs. Our group is now working on characterizing the biochemical and histological changes resulting from histamine-induced conjunctivitis in dogs.

In summary, histamine-induced conjunctivitis in dogs represents a robust and reliable model for translational research on the ocular surface. The model is particularly appealing given the low cost, non-invasiveness, self-resolving nature, ability to adjust the duration and severity of the disease, and shared features with naturally occurring diseases in human and veterinary medicine. The model could be used to assess new therapeutics and to better understand the impact of conjunctivitis on drug pharmacokinetics–pharmacodynamics, tear film dynamics, and tear film composition, among many other applications.

## Data Availability

The datasets generated for this study are available on request to the corresponding author.

## Ethics Statement

The study was approved by the Institutional Animal Care and Use Committee of Iowa State University (log # 2-18-8704-K) and adhered to the Association for Research in Vision and Ophthalmology (ARVO) statement for the Use of Animals in Ophthalmic and Vision Research.

## Author Contributions

LS conceptualized and designed the study in consultation with RA and JM. LS, RA, and AW performed the experiments. LS, Y-JS, and JM analyzed the data. All authors wrote the manuscript.

## Conflict of Interest Statement

The authors declare that the research was conducted in the absence of any commercial or financial relationships that could be construed as a potential conflict of interest.
