# Supplementary material for: Histamine-Induced Conjunctivitis and Breakdown of Blood–Tear Barrier in Dogs: A Model for Ocular Pharmacology and Therapeutics
Source: Front Pharmacol. 2019 Jul 9;10:752. doi: 10.3389/fphar.2019.00752 (PMC6629934; doi:10.3389/fphar.2019.00752)
Supplement: Supplementary file 1 [file Table_1.pdf]

Appendix A: Diagram of the study design showing the balanced crossover trial (top) and the timing of the procedures prior to and following topical histamine/vehicle administration (bottom). BP= Blood pressure; CS = Conjunctivitis scoring; IOP = Intraocular pressure.

	Day 1	Day 2	Day 3	Day 4	Day 5	Day 6
Dog 1	H_2	H_3	H_6	H_4	H_1	H_5
Dog 2	H_4	H_5	H_1	H_2	H_3	H_6
Dog 3	H_6	H_1	H_2	H_5	H_4	H_3
Dog 4	H_1	H_6	H_5	H_3	H_2	H_4
Dog 5	H_3	H_2	H_4	H_6	H_5	H_1
Dog 6	H_5	H_4	H_3	H_1	H_6	H_2

H_1 = 0.005 mg/mL

H_2 = 0.1 mg/mL

H_3 = 1.0 mg/mL

H_4 = 10 mg/mL

H_5 = 375 mg/mL

H_6 = 500 mg/mL

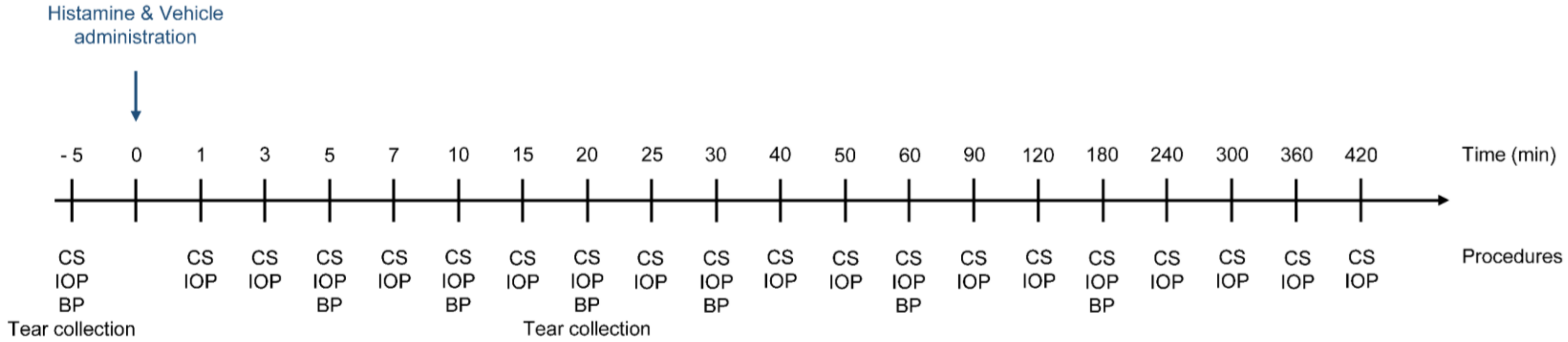

Appendix B: A semi-quantitative conjunctivitis scoring system.

	Score	Description
**Palpebral conjunctiva**		
Chemosis	0 (none)	No swelling of the palpebral conjunctival tissue.
	1 (mild)	Diffuse thin swelling with no eversion of the eyelid(s) or change in eyelid margin contour.
	2 (moderate)	Diffuse definite swelling with misalignment of the normal approximation of the lower and upper eyelids.
	3 (severe)	Diffuse marked swelling with partial eversion of the eyelid(s). The eyelid margin(s) may have an irregular, 'undulating' contour, but can still be closed completely.
	4 (very severe)	Extremely severe swelling with pronounced eversion of both eyelids. Eyelid closure is incomplete with exposed swollen conjunctiva protruding between the eyelid margins and masking the corneal surface.
Hyperemia	0 (none)	Small individual vessels are noted, blanched to pale pink in color. It is normal to observe a few prominent vessels on the palpebral surface of the third eyelid.
	1 (mild)	Dilatation of only a few vessels with minimal branching and/or tortuosity. Pink to light red in color.
	2 (moderate)	Dilatation of the majority of vessels with pronounced branching and/or tortuosity. Bright red to crimson red in color. The conjunctiva between large vessels may have a flushed pink-to-red appearance.
	3 (severe)	Diffuse beefy red appearance to the conjunctiva, difficult to distinguish individual blood vessels.
Follicles	0 (none)	No manifestations
	1 (mild)	1-9 follicles
	2 (moderate)	10-19 follicles
	3 (severe)	20 or more follicles
**Bulbar conjunctiva**		
Chemosis	0 (none)	No swelling of the bulbar conjunctival tissue.
	1 (mild)	Focal perilimbal and/or diffuse thin swelling. Underlying episcleral tissue is easily observed through the conjunctiva.
	2 (moderate)	Diffuse definite swelling. Underlying episcleral tissue is harder to observe.
	3 (severe)	Diffuse marked swelling, masking the corneoscleral limbal region and underlying episcleral tissue.
Hyperemia	0 (none)	Small individual vessels are noted, blanched to pale pink in color.
	1 (mild)	Dilatation of only a few vessels with minimal branching and/or tortuosity. Pink-to-reddish in color.
	2 (moderate)	Dilatation of the majority of vessels with pronounced branching and/or tortuosity. Bright red to crimson red in color. The conjunctiva between large vessels may have a flushed pink-to-red appearance.
	3 (severe)	Diffuse beefy red appearance to the conjunctiva, difficult to distinguish individual blood vessels.
Follicles	0 (none)	No manifestations
	1 (mild)	1-9 follicles
	2 (moderate)	10-19 follicles
	3 (severe)	20 or more follicles
**Conjunctival discharge**	0 (none)	No discharge, or small amount of clear/mucoid material found in the medial canthus.
	1 (mild)	Discharge is above normal and present on the surface of the eye or in the medial canthus, but not on the lids or hairs of the eyelids.
	2 (moderate)	Discharge is abundant, easily observed, and has collected on the lids and around the hairs of the eyelids.
	3 (severe)	Discharge has been flowing over the eyelids so as to wet the hairs substantially on the skin around the eyes, past the orbital rim.
**Ocular pruritus**	0 (none)	No ocular itching
	1 (mild)	Subtle, rapidly resolving itch
	2 (moderate)	Mild persistent itch, resolving within 30 seconds
	3 (severe)	Pronounced itch, not resolving within 30 seconds
	4 (extremely severe)	Incapacitating itch

Appendix C: Summary table detailing each subsection of the conjunctivitis score for each histamine dose. The score selected in each individual was the maximal score documented between 0 and 420 min following histamine administration. T_{max} describes the time (in min) to reach this maximal score in each dog. Results are described as mean ± standard deviation.

		Palpebral chemosis	Palpebral hyperemia	Palpebral follicles	Bulbar chemosis	Bulbar hyperemia	Bulbar follicles	Conjunctival discharge	Ocular pruritus
H_1 (0.005 mg/mL)	Dogs affected	0/6	6/6	0/6	1/6	3/6	0/6	0/6	0/6
	Score	0	1.2 ± 0.4	0	1	1.0 ± 0	0	0	0
	T_{max} (min)	0	4.7 ± 1.5	0	7	15 ± 10	0	0	0
H_2 (0.1 mg/mL)	Dogs affected	0/6	6/6	0/6	2/6	6/6	0/6	0/6	0/6
	Score	0	1.8 ± 0.4	0	1 ± 0	1.5 ± 0.5	0	0	0
	T_{max} (min)	0	4.5 ± 3.1	0	6.0 ± 1.4	4.3 ± 2.1	0	0	0
H_3 (1.0 mg/mL)	Dogs affected	2/6	6/6	0/6	1/6	6/6	0/6	0/6	2/6
	Score	1.0 ± 0	2.0 ± 0	0	1	2.0 ± 0	0	0	1.0 ± 0
	T_{max} (min)	5.0 ± 0	4.0 ± 2.1	0	25	6.8 ± 3.5	0	0	1.0 ± 0
H_4 (10 mg/mL)	Dogs affected	6/6	6/6	0/6	6/6	5/6	0/6	0/6	4/6
	Score	1.0 ± 0	2.0 ± 0	0	1.5 ± 0.5	2.0 ± 0	0	0	1.3 ± 0.5
	T_{max} (min)	5.2 ± 2.6	2.0 ± 1.1	0	6.5 ± 2.0	3.2 ± 3.9	0	0	1.0 ± 0
H_5 (375 mg/mL)	Dogs affected	6/6	6/6	0/6	6/6	6/6	0/6	1/6	6/6
	Score	2.2 ± 0.8	2.2 ± 0.4	0	2.5 ± 0.5	2.0 ± 0	0	1	2.5 ± 0.8
	T_{max} (min)	6.3 ± 4.5	2.0 ± 2.4	0	10.7 ± 5.8	1.0 ± 0	0	15	1.0 ± 0
H_6 (500 mg/mL)	Dogs affected	6/6	6/6	0/6	6/6	6/6	0/6	0/6	6/6
	Score	2.7 ± 1.0	2.0 ± 0	0	2.3 ± 0.8	2.0 ± 0	0	0	2.8 ± 0.4
	T_{max} (min)	9.2 ± 5.4	1.0 ± 0	0	11.2 ± 15.0	1.0 ± 0	0	0	1.0 ± 0
